# Adrenaline and return of spontaneous circulation during in-hospital cardiac arrest

**DOI:** 10.1016/j.resplu.2025.101140

**Published:** 2025-10-20

**Authors:** A. Norvik, E. Unneland, D. Bergum, J.P. Loennechen, J.T. Kvaløy, E. Aramendi, J. Urtega, E. Skogvoll

**Affiliations:** a Department of Circulation and Medical Imaging, Faculty of Medicine and Health Sciences, Norwegian University of Science and Technology (NTNU), Trondheim, Norway; b Department of Anesthesia and Intensive Care Medicine, St Olav University Hospital, Trondheim, Norway; c Clinic of Cardiology, St. Olav University Hospital, Trondheim, Norway; d Department of Mathematics and Physics, University of Stavanger, Stavanger, Norway; e University of the Basque Country, Engineering School of Bilbao, Bilbao, Spain

**Keywords:** In-hospital cardiac arrest, Adrenaline, Return of spontaneous circulation

## Abstract

**Introduction:**

Adrenaline provides inotropic, chronotropic and vasopressor effects and is a cornerstone drug during cardiopulmonary resuscitation. This study of in-hospital cardiac arrest aimed to investigate the effect of adrenaline on the return of spontaneous circulation (ROSC) in primary pulseless electrical activity (PEA).

**Method:**

In-hospital cardiac arrests episodes at St. Olav University Hospital (Norway) were prospectively registered between 2018 and 2022, among these were 73 episodes with primary PEA. Time of adrenaline administration was obtained with minutes precision by reviewing the clinical record and interviewing personnel, thereby establishing a consistent timeline. We investigated transitions from primary PEA to ROSC following adrenaline administration using time-to-event models.

**Results:**

Adrenaline exerted its maximum effect between 45 and 85 s after administration and strongly favored the transition from primary PEA to ROSC, with an intensity ratio of 5.03 (*p* < 0.001). Repeated doses of adrenaline, however, had no effect in case of absence of an initial response. Basic life support alone yielded a transition intensity from primary PEA to ROSC of about 0.06, i.e., 6 % per minute.

**Conclusion:**

We found a rapid and five-fold increase in the transition intensity from primary PEA to ROSC approximately one minute after adrenaline administration. Repeated doses in the absence of an initial response did not alone increase the transition probability.

## Introduction

Pulseless electrical activity (PEA) is highly prevalent during IHCA where approximately half of the population presents with this rhythm and 80 % of all patients with IHCA experiences PEA at some point during resuscitation.^1^, ^2^, ^3^ Intravenous adrenaline is recommended as soon as possible to all patients with non-shockable cardiac arrest (CA).^4^ Adrenaline has chronotropic, inotropic and vasopressor effects.^5^ It was suggested that increasing coronary perfusion pressure promotes return of spontaneous circulation (ROSC)^6^, ^7^ and that adrenaline may facilitate this. Its effect and role have been discussed for decades. Observational studies during the 90s and early 2000s actually showed worse outcome for patients receiving adrenaline.^8^, ^9^, ^10^, ^11^ These underestimated the effect of adrenaline as they did not account for “resuscitation time bias” when directly comparing patients who received adrenaline to those who did not.^12^, ^13^ Adrenaline is not administered at random. Patients with good prognosis are more likely to respond to basic life support (BLS) alone and achieve ROSC early, obviating the need for adrenaline. Therefore, one cannot deduce the effect of adrenaline in an observational study by simply comparing patients according to whether adrenaline was given or not. While favoring ROSC, adrenaline is also a marker of poor prognosis. Resuscitation time bias can be avoided in group-randomized trials, but also with time-to-event analysis if adrenaline is treated as a time dependent covariate as this is a “forwards” approach.^14^ Randomized trials in out-of-hospital cardiac arrest (OHCA) have established that adrenaline increases the probability of ROSC^15^, ^16^, ^17^. Although the effect was small, Perkins et al. also found increased 30-day survival among the 4015 patients receiving adrenaline during OHCA.^17^ No randomized controlled trial (RCT) has been conducted to explore the effect of adrenaline in IHCA.^18^ The effect cannot fully be inferred from prehospital data, which is dominated by initial asystole, substantial differences in etiology, and late administration of adrenaline. Time to treatment is very important for survival after CA; delayed CPR and delayed defibrillation reduce survival.^19^, ^20^ Observational studies on IHCA have concluded that delayed adrenaline administration also reduces survival, but in a sub analysis of the PARAMEDIC2 trial one could not find a significant difference in long-term survival and neurological outcome between patients receiving early adrenaline or placebo.^19^, ^21^, ^22^

The aim of this study of adrenaline in primary PEA on return of spontaneous circulation was to describe how quickly adrenaline works and to quantify its effect. Secondly, we estimated the effect of basic life support alone in the absence of adrenaline.

## Methods

### Study setting and population

This prospective observational study was conducted between August 2018 and October 2022 at St. Olav University Hospital in Trondheim, Norway. This tertiary care facility covers all medical specialties, with approximately 900 beds, 60 000 admissions per year, and an IHCA incidence of about 1.3–1.7 per 1000 admissions yielding 80–100 episodes per year. All hospital employees are trained in basic life support (BLS). In the event of CA, ward personnel immediately initiate BLS and call the emergency team (ET). The ET consists of a cardiologist, an anesthesiologist, a nurse anesthetist, and an orderly. The first i.v. dose of adrenaline (usually 1 mg) is given according to international ALS guidelines^23^ by the ET or by an attending physician in the intensive care units (ICU) as soon as the situation allows. The sickest patients may be in ICUs or step-down units that are close to the ET base, otherwise there is no relation between illness severity and distance to patient location (e.g., wards, radiology labs). A defibrillator (LifePak 20 or LifePak 1000, Physio-Control, Redmond, USA) records the ECG, the transthoracic impedance, and any shocks delivered. Events like intubation, adrenaline administration, etc. are registered manually by the ET or automatically by the defibrillator.

### Data collection and processing

The defibrillator file and the patient’s clinical records were reviewed to determine the course of events; the presenting rhythm, chest compressions, and whether (and when) adrenaline was administered. If the timing of adrenaline had not been documented, we interviewed personnel present at the event, linking and reconciling the information with other registered events (e.g., a DC shock, change of defibrillators longer pauses in chest compression etc.).

Each defibrillator file was analyzed in a graphical tool developed in Matlab® as described in earlier publications.^2^, ^24^ Briefly, the first recording of ECG marked the beginning of the episode (defibrillators are widely available and attached to the patient shortly after the collapse). From the ECG and transthoracic impedance signals we noted the evolution of clinical states (asystole, PEA, VF/VT, ROSC) along the timeline of the episode, including times of adrenaline administration. Due to noise in the ECG signal during compressions, the clinical state was only analyzed during compression pauses. Primary PEA was defined when PEA was the presenting rhythm, lasting until a transition to any other clinical state (ROSC, Asystole, VF/VT, Death). ROSC was defined as a cardiac rhythm compatible with spontaneous circulation and distinguished from PEA if chest compressions were paused for longer than one minute as reflected in the impedance signal and in agreement with clinical recordings. This definition would thus include both temporary (>1 min and <20 min) as well as sustained ROSC (>20 min).

### Statistical modelling

Using different time-to-event models, we investigated the relation between administration of adrenaline and transitions from primary PEA to ROSC as the outcome. In this multistate model, transitions to other clinical states than ROSC (including death declared, asystole and ventricular tachycardia/fibrillation) were treated as censoring.

**Fig. 1:**
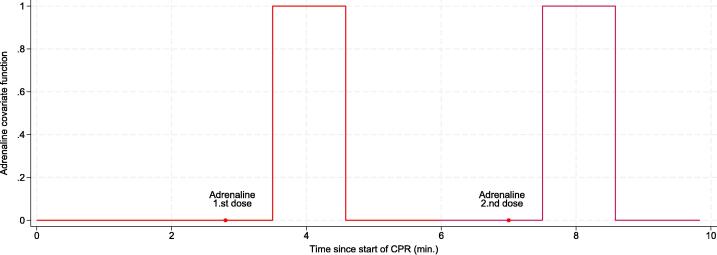
Example of an adrenaline covariate profile. The red dot represents the time of adrenaline administration, and the red line represents the time dependent covariate value changing from 0 to 1 and from 1 to 0 at a given timepoint after administration. (For interpretation of the references to colour in this figure legend, the reader is referred to the web version of this article.)

We estimate the effect of adrenaline as the intensity ratio (IR, otherwise often known as “hazard ratio” in time-to-event models), by comparing the number of observed transitions to ROSC shortly after adrenaline to transitions occurring at other times. While adrenaline must reach the central circulation to work, the plasma concentration after administration was not measured in this study. As a simple approximation we created a time-dependent covariate (“AD”) along the timeline, as follows: prior to adrenaline administration AD would be 0, then change to 1 when adrenaline might begin to exert its effect, remain at 1 for some time (i.e., a “window of opportunity”), and return to 0 when this “window” closes. By splitting each episode into 5 s intervals one can capture covariates that change over time. However, as neither the time of onset nor the duration of the window of opportunity was well known a priori, we tried a total of 171 different combinations of onset and duration to find the best fit: onset from 0 to 90 s after administration in 5 s steps, and duration between 10 to 90 s after the onset in 10 s steps. This was done for the 1st, 2nd and 3rd dose. Fig. 1 shows one example of AD with an onset at 45 s and a window of 60 s, i.e. from 45 to 105 s after administration. We compared the different candidate AD profiles with respect to model fit using Cox regression models with Akaike’s Information Criterion (AIC) as goodness of measure. Finally, we chose the AD profile with the best fit for further analysis.

To separate the effect of adrenaline from the effect of basic life support alone, we employed parametric time-to-event models that allow for estimation of the basic transition intensity in addition to the effect of covariates. We used proportional intensity models, both an exponential model assuming constant intensity, and a Greenwich model that allows for a unimodal (i.e., an increasing and then decreasing) intensity function.^25^

In addition to giving adrenaline, the emergency team also provides senior clinical support and interventions like airway management, respiratory support, and diagnostics. To check whether ET arrival time on scene independently also impacted on transitions to ROSC or acted as a confounder, we created an ordinal ET response time variable with 6 levels. The classification was based on the distance between predefined checkpoints en route to the patient from the ET base, considering known delays and obstacles (e.g., crossing an internal bridge, calling the elevator). This covariate was added to the model, both as continuous and as categorical. Finally, we checked whether the level of care (Operating room/ intensive care unit (OR/ICU), intermediate care unit/ emergency department, or general ward/radiology lab. etc.) had any impact.

### Ethical considerations

The Regional Committees for Medical and Health Research Ethics in central Norway approved the study (reference number 2019/785). The need for consent was waived by the committee for patients included after June 2019. The remaining patients provided written consent personally or through a next-of-kin.

## Results

In total, we registered 287 episodes of CA in 266 patients and their characteristics are summarized in Table 1.

**Table 1:** Description of the cardiac arrest population and the included patients. Intra-aortic balloon pump (IABP), Pulseless electrical activity (PEA), Ventricular fibrillation/tachycardia (VF/VT), return of spontaneous circulation (ROSC), extracorporeal membrane oxygenation (ECMO).

**Cardiac arrest population**	
Patients	266
−Mean age (min,max)	70.0 years (22,97)
−Female gender	92 (32.7 %)
−Survived to discharge	114 (44.2 %)
Episodes and their primary rhythm	287
−PEA	96 episodes (33.4 %)
−Asystole	43 episodes (15.0 %)
−VF/VT	130 episodes (45.3 %)
−Unknown initial rhythm	17 episodes (5.9 %)
−Adrenaline administration	123 episodes (45,7%)
Outcome of episodes	
−ROSC	183 episodes
−ECMO	4 episodes
−IABP	1 episode
**Included episodes with PEA as primary rhythm**	
Included in analysis	73
−Female (survived to discharge)	25 (7)
−Male (survived to discharge)	48 (7)
−Median age	71 years
−Assumed cardiac etiology	29
−Median time to adrenaline (IQR)	3 min 5 s (1 min 20 s – 5 min 10 s)
−Median time in primary PEA (IQR)	4 min 5 s (2 min 15 s – 9 min)
State following primary PEA	
−ROSC	35
−VF/VT	8
−Asystole	12
−Declared dead	15
−Censored in primary PEA	1
Outcome of episode	
−Sustained ROSC	37
Precision of time estimate 1st dose	
Exact	14
+/- 30 s	16
+/- 60 s	4
+/- 90 s	3
Precision of time estimate 2nd dose	
Exact	11
+/- 30 s	8
+/- 60 s	0
+/- 90 s	2
Precision of time estimate 3rd dose	
Exact	7
+/- 30 s	5
+/-60 s	0
+/- 90 s	1

From the 96 episodes with PEA as presenting rhythm, we excluded 23 episodes (14 due to missing defibrillator files, six because of missing information about the time of adrenaline administration, 2 due to ongoing adrenaline infusion during resuscitation and 1 due to thoracotomy during resuscitation); leaving 73 episodes for analysis. All doses of adrenaline were administered intravenously. Descriptive characteristics of the episodes are summarized in Table 1. The median time to adrenaline administration was 3 min (Range 0.02 to 10.97 min) after attachment of the first defibrillator (done shortly after the collapse, as all wards have access to an AED). The location of patients and the presumed etiology is shown in Table 2.

**Table 2:** Overview of etiology and location of patients.

**Etiology**	**Ward**	**Intermediary department**	**Intensive care unit**	**Total**
Non-cardiac	18	5	8	31
Cardiac	13	3	12	28
Unknown	9	3	2	14

### Overall effect of the first dose of adrenaline in primary PEA

**Fig. 2:**
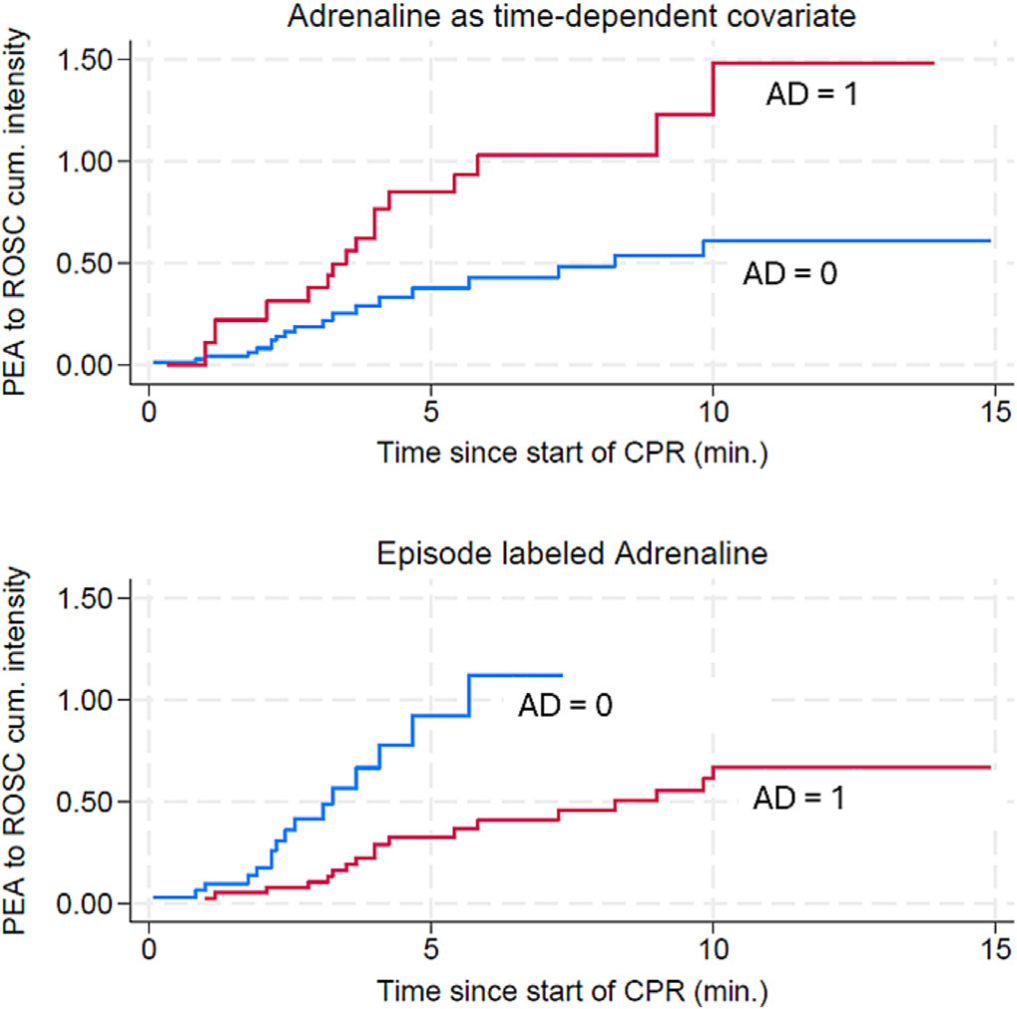
The cumulative intensity of transitioning to ROSC in patients where AD is 1 (red) and AD is 0 at the particular time. Upper panel: Adrenaline is included as a time dependent variable allowing the individual patient to change group from 0 to 1 and back to 0 as time passes. The population is stratified according to whether we believe adrenaline exerts its effect or not at the given time. Patients in primary PEA have a higher probability of achieving ROSC when adrenaline is administered. Lower panel: Adrenaline is included as a static variable and AD remains constant keeping the individual patient within the same group over the course of time stratifying the population according to whether they received adrenaline or not. The plot illustrates how resuscitation time bias would affect the result. When we do not account for the time dependency of adrenaline, it appears as if the patients do better without adrenaline. (For interpretation of the references to colour in this figure legend, the reader is referred to the web version of this article.)

Fig. 2 shows the timely development of ROSC during resuscitation as plots of cumulative intensity, according to whether AD was 1 or 0. The upper plot, where adrenaline is properly included as a time-dependent covariate (reflecting when we know adrenaline is present in the patient’s circulation), shows a strong and almost immediate effect of adrenaline. The lower plot shows the result when adrenaline is erroneously included as an episode-fixed variable.

### How quickly did adrenaline work, what is the effect and how does it impact on the transition intensity?

**Fig. 3:**
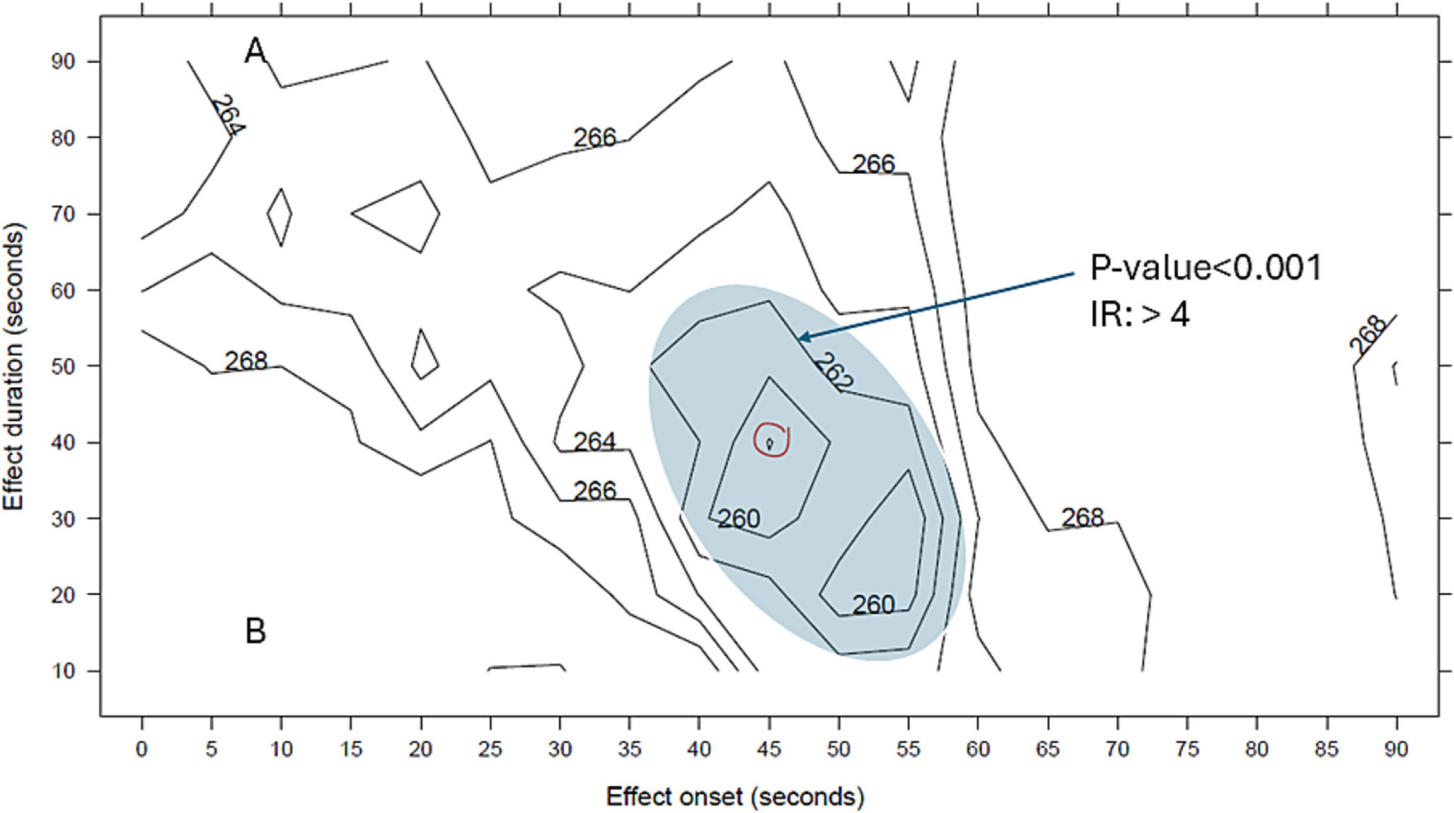
The contour plot illustrates the AIC-values of the Cox-models according to different effect onsets (x-axis, 0 to 90 s) and effect durations (y-axis, 10 to 90 s). The model with the best fit to our data has an effect onset of 45 s and an effect duration of 40 s with an AIC value of 257.9 (circled). Thus, the transition to ROSC often occurred 45–85 s after adrenaline administration. The shaded area highlights alternative models with low AIC values, p-values less than 0.01, and intensity ratios greater than 4, suggesting that the window may be broader than indicated by the best-fitting model.

We compared the different AD candidate profiles by inspecting the bivariate AIC plot and noted that ROSC was most likely to occur in a time window from 45 to 85 s after administration of the first dose, i.e., onset at 45 s and an effect window of 40 s (Fig. 3).

**Fig. 4:**
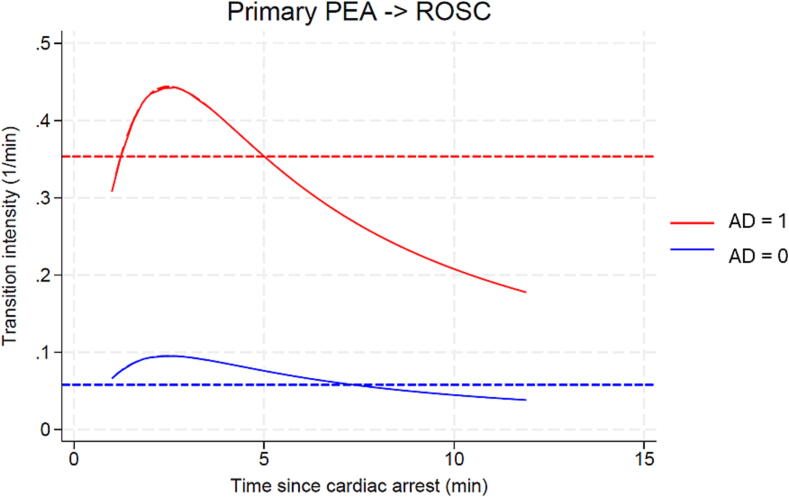
The changes in intensities over time for the first 12.5 min of CPR using the Greenwich model (full line). This allows us to separate the effect of basic life support from the effect of adrenaline. The exponential model (dashed line) provides a constant estimate of the intensity rate over time. According to the Greenwich model a patient in primary PEA 2.5 min into resuscitation may increase the chances of achieving ROSC approx. 3.5 times if adrenaline is administered (AD changes from 0 to 1). The exponential model estimates a 6 fold increase.AD (Time dependent adrenaline covariate).

By the Cox semiparametric model, we estimated an intensity ratio of 5.03 (95 % CI 2.16 to 11.67). By the parametric exponential and Greenwich regression models, the intensities over time for the first 15 min of CPR are plotted in Fig. 4, again showing an intensity ratio of the same magnitude (exponential model: 6.07, 95 % CI 2.78 to 13.29; Greenwich model: 4.66, 95 % CI 2.13 to 10.19). Basic life support alone resulted in an intensity of 0.06 for the PEA to ROSC transition according to the exponential model (constant over time), i.e., approximately 6 % per minute of CPR. The more physiologically adapted Greenwich model (allowing for varying intensity over time) indicated that the transition intensity peaked at about two to three minutes from the start of CPR, which is consistent with earlier results.^2^

We found no significant effect of the time of emergency team arrival, neither as a main effect nor as an interaction with adrenaline, nor with the level of care.

As a brief sensitivity analysis regarding the choice of covariate profile, we extended the time window to 60 and 90 s and observed intensity ratios of 3.7 (*p* = 0.002) and 2.6 (*p* = 0.02), respectively; suggesting a dilution of effect, as expected.

### The second and third dose of adrenaline

We observed no association between the 2nd and the 3rd dose of adrenaline given in primary PEA, and the transition to ROSC in 22 and 13 episodes, respectively.

## Discussion

The main finding in this study of in-hospital cardiac arrest with primary PEA is that adrenaline exerts its effect within 60 seconds after administration and increases the return of spontaneous circulation three to five-fold when compared to basic life support alone. Although it is known that adrenaline increases the probability of ROSC,^15^, ^16^, ^17^, ^18^ the details surrounding the actual transition to ROSC in IHCA have, to our knowledge, not been described before.

May we assume a causal relation between adrenaline and the return of spontaneous circulation? We believe that this is reasonable, because of the longitudinal design that separates intervention and response, and because the time of adrenaline administration was registered to minutes precision. Our focus on the first minutes of resuscitation makes interference from other, more advanced resuscitative efforts less likely (e.g. intubation, thrombolysis, and specific measures to reverse the cause), and we found no evidence of this when including the time to ET arrival into the analysis. Furthermore, in a secondary analysis of the randomized trial of i.v. medication or not (in practice, adrenaline) in out-of-hospital CA, Nordseth and colleagues showed,^26^ (Fig. 4d) that the PEA to ROSC transition intensity was approximately 3 times higher with adrenaline. This observation fits well with our results and supports our notion of causality. Also our findings are consistent with the results of Heradstveit et al.,^27^ who sampled blood from the jugular vein in 9 patients every minute after administration of adrenaline in a peripheral vein of patients with OHCA, and found a peak adrenaline concentration one min after administration with a half-life of 2.6 min.

Adrenaline must reach the central circulation to have effect, so the circulation of adrenaline may be determined by patient characteristics (i.e., residual circulation, or “pseudo-PEA) or the quality/ effectiveness of the chest compressions given (compression point, depth, or rate). This study suggests that adrenaline is circulated quickly by ongoing chest compressions. It is also possible that a smaller dose of adrenaline might have been equally effective, something that ought to be investigated. Patients receiving adrenaline may either transition to ROSC, transition to other arrest states (asystole, VF/VT), or not respond (i.e., remain in PEA). A study by Donnino et. al. found that shorter time to adrenaline was associated with higher probability of ROSC, in-hospital survival and survival with good neurological function in patients with non-shockable IHCA.^13^ While early i.v. adrenaline would probably benefit the adrenaline responders, this may be at the expense of those patients with a good prognosis who only need BLS to gain ROSC.

In this study, the non-responders remaining in primary PEA after the 1st dose often received a second and a third dose, but we found no effect of these. Poor chest compression technique or specific patient characteristics might have precluded an effect in the first place. The chest compression technique might be improved at a later stage (e.g., changing compression point, depth or rate), potentially circulating the adrenaline centrally to promote ROSC. Such an effect would be difficult to find within the framework of our study. In our study, there were few available episodes to analyze, and we cannot conclude with absolute certainty whether the 2nd and the 3rd doses were ineffective or not. We may speculate, however, that those who respond may have had some residual circulation and that non-responders constitute a different patient population.

In total, 38 % of those receiving adrenaline achieved sustained ROSC. Surprisingly, this is comparable to ROSC rates in the adrenaline arms of the three RCTs (25 %, 36 % and 40 %) OHCA.^15^, ^16^, ^17^ In the work of Donnino et al. the in hospital sustained ROSC rate was 49 %.^13^ We expected that the immediate in-hospital availability of adrenaline would lead to even higher rates of ROSC, since early administration of adrenaline is associated with higher survival.^13^, ^21^, ^28^, ^29^

Our study also demonstrates the importance of handling time-dependent covariates properly. A potential misinterpretation of the effect of adrenaline will result if it is not properly treated as a time-dependent covariate. Merely labeling an episode by whether adrenaline was given or not leads to confounding by indication, and the effect will show up negative as illustrated in Fig. 2. This seemingly contradictory result appears because adrenaline only can exert its effect within a certain timeframe of an episode (e.g., only after administration) and because adrenaline is not administered at random (as patients transitioning quickly to other states are different from those who remain stable in primary PEA and eventually receive adrenaline). The phenomenon is also known as resuscitation time bias, akin to immortal time bias, where ROSC cannot occur before after the intervention, which in this case is adrenaline.^12^

### Limitations

This study has notable limitations as well as strengths.

The emergency team provides other interventions like airway management and clinical support. As team arrival is closely linked to administration of adrenaline, we cannot exclude the possibility of residual confounding as some other interventions also might have worked despite attempting to control for that in the analysis.

Without an objective measure of circulation, we do not know for sure whether the patient had actually achieved ROSC or not. Still, it is unlikely that the attending clinicians would interrupt chest compressions in a patient without circulation for a longer period. Dewolf et al. investigated interruptions during CPR and found that only 4.3 % were longer than 1 min, adding to the validity of our definition.^30^ It is difficult to estimate whether our definition over or underestimates ROSC; some patients also may have had palpable pulse for less than a minute.

We acknowledge some uncertainty with respect to adrenaline administration. Table 1 summarizes this, but we consider this to be minor. Also, ROSC is usually declared later than it actually occurs as one must pause chest compressions to assess the circulation and pulse may go unrecognized.

ROSC is obviously necessary for long-term survival, so while long-term survival is the ultimate outcome of interest this is influenced by an array of factors both related and unrelated to treatment provided during the arrest itself.

Finally, the number of episodes with primary PEA was limited, impairing the precision of the estimates.

A notable strength of the study is the well-defined timeline that allows for proper causal inference. Moreover, parametric time-to-event models allow separating the effect of BLS from that of adrenaline.

## Conclusion

We found a five-fold increase in transition intensity from primary PEA to ROSC after adrenaline administration. The transition may be expected within approx. 90 s of administration of adrenaline. Repeated doses seem futile in the absence of an initial response.

## CRediT authorship contribution statement

**A. Norvik:** Writing – review & editing, Writing – original draft, Methodology, Investigation, Formal analysis, Data curation, Conceptualization. **E. Unneland:** Writing – review & editing, Data curation, Conceptualization. **D. Bergum:** Writing – review & editing, Data curation, Conceptualization. **J.P. Loennechen:** Writing – review & editing, Data curation, Conceptualization. **J.T. Kvaløy:** Writing – review & editing, Formal analysis, Conceptualization. **E. Aramendi:** Writing – review & editing, Software, Data curation. **J. Urtega:** Writing – review & editing, Software, Data curation. **E. Skogvoll:** Writing – review & editing, Writing – original draft, Validation, Supervision, Project administration, Methodology, Investigation, Funding acquisition, Formal analysis, Data curation, Conceptualization.

## Declaration of competing interest

The authors declare that they have no known competing financial interests or personal relationships that could have appeared to influence the work reported in this paper.
